# Exercise alters the immune profile in Tg2576 Alzheimer mice toward a response coincident with improved cognitive performance and decreased amyloid

**DOI:** 10.1186/1742-2094-5-13

**Published:** 2008-04-09

**Authors:** Kathryn E Nichol, Wayne W Poon, Anna I Parachikova, David H Cribbs, Charles G Glabe, Carl W Cotman

**Affiliations:** 1 Institute for Brain Aging & Dementia, University of California, Irvine. Irvine, CA, USA; 2 Department of Molecular Biology & Biochemistry, University of California, Irvine. Irvine, CA, USA; 3 Department of Neurology, University of California, Irvine, CA, USA

## Abstract

**Background:**

Inflammation is associated with Aβ pathology in Alzheimer's disease (AD) and transgenic AD models. Previously, it has been demonstrated that chronic stimulation of the immune response induces pro-inflammatory cytokines IL-1β and TNF-α which contribute to neurodegeneration. However, recent evidence has shown that inducing the adaptive immune response reduces Aβ pathology and is neuroprotective. Low concentrations of IFN-γ modulate the adaptive immune response by directing microglia to differentiate to antigen presenting cells. Our objective was to determine if exercise could induce a shift from the immune profile in aged (17–19 months) Tg2576 mice to a response that reduces Aβ pathology.

**Methods:**

TG (n = 29) and WT (n = 27) mice were divided into sedentary (SED) and exercised (RUN) groups. RUN animals were provided an in-cage running wheel for 3 weeks. Tissue was harvested and hippocampus and cortex dissected out. Quantitative data was analyzed using 2 × 2 ANOVA and student's t-tests.

**Results:**

IL-1β and TNF-α were significantly greater in hippocampi from sedentary Tg2576 (TG_SED_) mice than in wildtype (WT_SED_) (p = 0.04, p = 0.006). Immune response proteins IFN-γ and MIP-1α are lower in TG_SED _mice than in WT_SED _(p = 0.03, p = 0.07). Following three weeks of voluntary wheel running, IL-1β and TNF-α decreased to levels indistinguishable from WT. Concurrently, IFN-γ and MIP-1α increased in TG_RUN_. Increased CD40 and MHCII, markers of antigen presentation, were observed in TG_RUN _animals compared to TG_SED_, as well as CD11c staining in and around plaques and vasculature. Additional vascular reactivity observed in TG_RUN _is consistent with an alternative activation immune pathway, involving perivascular macrophages. Significant decreases in soluble Aβ_40 _(p = 0.01) and soluble fibrillar Aβ (p = 0.01) were observed in the exercised transgenic animals.

**Conclusion:**

Exercise shifts the immune response from innate to an adaptive or alternative response. This shift in immune response coincides with a decrease in Aβ in advanced pathological states.

## Background

Successful long-term treatment for Alzheimer's disease (AD) has remained as elusive as the disease's cause. Lifestyle treatments for AD, such as exercise, are being researched in both animal and human models [1-5]. Exercise has been found to be a successful preventative measure in delaying disease onset. Exercise interventions in patients who already exhibit AD symptoms have had mixed results, but are able to show improvement to varying degrees [2-5].

The pathology in AD is well-known. Aβ and tau deposits are the chief pathological hallmarks. Aβ has been implicated in impairing learning and memory [6-10]. Aβ deposition stimulates a local immune response by the microglia, which become macrophagic [11-13]. The macrophagic phenotype in AD is characterized by the presence of CD11b and immunohistochemistry revealing attempted phagocytosis of Aβ [11,14-16]. This response is not sufficient to clear Aβ deposits and instead contributes to the chronic progression of AD [17-19]. Chronically activated microglia are characterized by their macrophagic morphology, CD11b expression, and release of neurotoxic cytokines IL-1β and TNF-α [20,21]. This response of microglia seems to contribute to AD progression, rather than clearing Aβ pathology [22-24].

However, the immune responses possible in the brain are more complex than was once thought. Aside from the initial innate response, characterized by macrophagic microglia and cytokine production, adaptive responses in which antigen is presented to T cells infiltrating from the periphery has been increasingly observed in various neurodegenerative disease states and in Aβ vaccination studies [14,25-28]. In addition, exciting new evidence exists of an alternative immune response, characterized by perivascular cells that participate in both innate immunity, via phagocytosis and cytokine production, as well as adaptive immunity via antigen presentation and co-stimulation at the blood brain interface [29-31]. Evidence indicates that the AD brain is capable of clearing Aβ if the microglial response is manipulated [32]. For instance, injected bone marrow cells travel to the brain and differentiate into microglia. These newly differentiated microglia acquire a dendritic, rather than macrophagic phenotype and are associated with antigen presentation and a decrease in Aβ [32]. Further, microglia stimulated with glatiramer acetate or IFN-γ change phenotype and become antigen presenting cells [33-35]. This antigen presenting phenotype stimulates the adaptive immune response, characterized by increased major histocompatibility complex II (MHCII) expression and CD11c positive cells [34,36]. Butovsky et al.(2007) and Ziv et al (2006) have also shown this shift to the adaptive immune response coincides with decreases in cytokines associated with the innate immune response, namely IL-1β and TNF-α [34,37]. The identity of the antigen presenting cells was initially unclear in these studies. However, it has recently been suggested that the clearance of Aβ is dependent on invasion of the brain by peripheral monocytes, which differentiate into antigen presenting microglia [35,38]. A stimulus to the immune response in late AD may confer benefit through clearance of soluble species of Aβ, as evidenced in several studies using lipopolysaccharide or Aβ immunization [39-41]. The role of microglia in AD is clearly complex and can be either detrimental or beneficial, depending on the nature of the microglial response.

While physical exercise can delay the onset of cognitive decline in AD, the mechanisms remain largely unknown [42-46]. Adlard et al. recently demonstrated that exercise reduced Aβ deposition in AD transgenic mice [1]. We suggest that exercise may be also altering the immune response in AD, which in turn affects Aβ levels. We hypothesize that the protective effects of exercise in AD may be due in part to an immune shift from innate to antigen presenting, similar to the shift observed by Butovsky et al (2005–2007) [33,35]. Specifically, we hypothesize that this shift is characterized by decreases in markers of the innate response, such as TNF-α and IL-1β, as well as increases of adaptive and alternative activation response stimulating cytokine IFN-γ. We further suggest that exercise induces a microglial phenotype shift from a macrophagic phenotype to an antigen presenting phenotype, expressing CD11c and MHC II. We also expect to see increased CD40, which communicates from the antigen presenting cell to CD40 ligand on T cells. It is possible this shift in immune response could decrease Aβ species in the Tg2576 mouse, as seen in the Butovsky studies [33-35,38]. In contrast to Butovsky, our study is unique in that we examined whether physical exercise can induce this immune shift without pharmacological intervention.

In the current study, we used the Tg2576 AD mouse model developed by Karen Hsiao [47]. Tg2576 mice exhibit plaque pathology at 10 months, but show cognitive deficits as early as 6 months [8,48]. For the present study, we were interested in using aged mice with advanced pathology and memory deficits. The older mouse with advanced pathology serves as a model for later stages of AD. We have previously demonstrated that three weeks of voluntary running improved water maze performance in aged (17–19 month) Tg2576 mice [49]. In this investigation, we examine a shift in the immune response to Aβ as a potential mechanism for the behavioral improvements.

## Methods

### Animals

The University of California, Irvine's Institutional Care and Use Committee approved all animal protocols. We used C57Bl6/SJL (WT) and Tg2576 (TG) from an established colony (ca. 2000) at University of California, Irvine. We assigned Tg2576 (n = 29) and C57Bl6/SJL (n = 27) to cages containing exercise wheels (RUN) or size matched cages without wheels (SED) at 16–18 months of age, singly-housed. We provided RUN animals with running wheels for 3 weeks and monitored their running via software (Minimitter).

We sacrificed RUN animals along with SED animals by decapitation. We then dissected hippocampi and cortices in physiological saline solution (145 mM NaCl, 4.7 mM KCl, 2 mM CaCl, 1 mM MgSO_4_, 4 mM MOPS, 1 mM NaH_2_PO_4_, 5 mM glucose, 2 mM pyruvic acid, 20 μM EDTA; pH 7.4), placed in microfuge tubes on dry ice, and stored frozen at -80°C. Frozen tissue was prepared by pulverization on dry ice. Pulverized tissue was soniccated in cell lysis buffer (Bio-Rad) according to Hulse et al. [50]. Protein content determined by bichondruric acid method (Bio-Rad, Hercules, CA).

### Cytokines

Bio-plex multi-plex analysis (Bio-Rad, # X600063YDF) was performed according to manufacturer's instructions in a custom kit designed to detect multiple cytokines. The bio-plex technology uses polystyrene beads internally dyed with differing ratios of two spectrally distinct fluorophores. Dyed beads are labeled with antibodies for each cytokine and the antibody-conjugated beads are allowed to react with sample and a secondary antibody in a 96-well plate to form a capture sandwich immunoassay. The assay solution is read by a Bio-Plex array reader, which distinguishes the beads' spectral address. Using the amount of green fluorescence emitted by the phycoerythrin-tagged detection antibody, the array reader extrapolates the concentration to the appropriate standard curve. Multiplex technology allows us to look at many cytokines within a single sample of tissue. We performed bio-plex analysis on 24 animals (n = 6 per group). We validated bio-plex findings for IFN-γ (BD Biosciences) and TNF-α (BD Biosciences) by ELISA. Because IL-1β levels were below detection sensitivity of the bio-plex assay, we also performed an ELISA for IL-1β, using this data in statistical analysis (Pierce).

### Western blot

IFN-γ activates an antigen presenting phenotype in microglia [34]. To further examine antigen presenting cell activity suggested by the cytokine profile observed in TG_RUN _vs. TG_SED_, we examined CD40 and MHC II protein expression in the hippocampus via western blot. For CD40 detection, 15 μg of hippocampal lysates from a randomly selected subset of animals (n = 8 TG_RUN_, n = 7 TG_SED_, n = 4 WT_RUN_, n = 9 WT_SED_) were prepared. Samples were prepared in loading buffer and boiled 5 minutes, then electrophoresed on 10% Tris-HCl ready-made gels (Bio-Rad) along with a homogenized liver sample as a positive control. Proteins were transferred to PVDF membrane overnight at 4°C. We blocked the membranes for 2 hours in StartingBlock™ PBS buffer (Pierce). We incubated membranes overnight at 4°C in 1:200 CD40 monoclonal antibody (BD Pharmingen, #550285) washed, and incubated in HRP-conjugated secondary anti-rat antibody (Santa Cruz, #sc-2065) 2 hours at 1:5000 in tris buffered saline with 1% Tween-20. We washed the membranes extensively, then used West Pico chemiluminescence to detect antigens. Results were visualized on Pierce CL-Xposure™ blue film. Results were quantified using Image J freeware as percent reactivity of CD40/β-actin (AbCam) and t-tests between WT_SED _and TG_SED_, as well as TG_SED _and TG_RUN _were performed.

Blots for MHC-II (1:100 BD-Pharmingen, #553538) were run on TG_SED _(n = 8) and TG_RUN _(n = 8) following the same procedure, using HRP-conjugated donkey anti-rabbit secondary antibody (1:1000, Jackson). Densitometric values for MHC II were normalized to β-actin (1:1000, SIGMA). Iba-1 (WAKO) was also used (1:2000) to probe the membranes for general microglia reactivity using the same basic paradigm. Iba-1 was quantified relative to β-actin.

### Aβ ELISA

It is not clear if an increase in adaptive immunity in the hippocampus would lead to changes in levels of the pathological hallmark of AD, Aβ, in animals of such advanced age. Cortical samples from TG_SED _(n = 5) and TG_RUN _(n = 6) were pulverized and then sonnicated in T-PER buffer (PIERCE) (10 μl/mg tissue). Samples were centrifuged for one hour at 100,000 g. Supernatant was removed and saved as the soluble fraction at -80°C. The insoluble fraction was resuspended in 70% formic acid and sonnicated. It was then spun a second time at 100,000 g for one hour and the formic acid layer (middle layer) removed and stored at -80°C.

Brain samples were run in triplicate on 96-well Immulon 2-HB plates (Thermo Electron Corp., Milford, MA) coated with a monoclonal anti-Aβ1–16 antibody at 25 ug/ml (kindly provided by Dr. William Van Nostrand, Stony Brook University, Stony Brook, NY) and detection was by monoclonal HRP-conjugated antibodies anti-Aβ_1–40 _at 1:500 (MM32-13.1.1) and anti-Aβ_1–42 _at 1:1000 (MM40-21.3.1) (kindly provided by Dr. Christopher Eckman, Mayo Clinic Jacksonville, Jacksonville, CA). Detection antibodies were visualized using the HRP-substrate 1-Step Ultra TMB-ELISA buffer (Pierce, Rockford, IL). For standards, synthetic Aβ_1–40 _and Aβ_1–42 _(Bachem California, Inc., Torrance, CA) were used after a pretreatment with HFIP to prevent fibril formation. Synthetic Aβ_1–40 _or Aβ_1–42 _were prepared in the same buffer as the samples and Aβ values were determined by comparison to the appropriate standard curve. The inclusion of a series of controls to test the absorbance of buffers, samples, and both capture and detection antibodies yielded negative results.

### Aβ dot blots & multiplex

Aβ species were quantified by dot-blot analysis. Hippocampal lysates were prepared from TG_SED _(n = 5) and TG_RUN _(n = 5) as described [8]. Wildtype samples (n = 2) were used as negative controls. Protein concentrations were determined by BCA (Pierce). An equal amount of protein from each sample was spotted onto nitrocellulose membranes (Schleicher and Schuell, 0.2 micron) and membranes were blocked 90 minutes at room temperature and incubated with appropriate primary antibodies (6E10 Covance, Princeton, NJ) and OC (kindly provided by Charles Glabe) 60 min at room temperature. These antibodies were used to assess total and fibrillar oligomeric Aβ, respectively. The OC antibody is conformation specific (for a more detailed description, see Kayed et al, 2007) [51]. Membranes were then washed, incubated with appropriate HRP-conjugated secondary (EMD Chemicals, San Diego, CA) and visualized by ECL (Supersignal West Pico, Pierce). Films were scanned and amyloid levels were quantitated using NIH Image J Software.

Aggregated Aβ was also measured in the aforementioned hippocampal samples and serum samples using multiplex technology and the human aggregated beta amyloid kit from Biosource/Invitrogen (catalog #LHB3491). The antibodies contained in this kit are specific for the 12 subunit oligomeric form of Aβ (also known as Amyloid Derived Diffusible Ligand, ADDL, Aβ*

### Immunohistochemistry

A small number of animals (n = 4 TG_SED_, n = 4 TG_RUN_, n = 4 WT_SED_, n = 4 WT_RUN_) were perfused with paraformaldehyde and brains dissected for immunohistochemistry for general microglia (CD11b) and dendritic (CD11c) microglial markers colocalized with Aβ (CD11b, BD Pharmingen; CD11c, Antigenix; Aβ, 6E10, Covance). Macrophagic markers CD68 (DAKO) and perivascular macrophage marker mannose receptor (HyCult Biotechnology) were used to identify macrophages and their relationship to CD11c+ cells and/or Iba-1 + microglia.

## Results

No significant differences in running distances existed between genotypes. (Table 1)

**Table 1:** Swim speeds and wheel rotations. Swim speeds did not significantly differ between any groups. Number of wheel rotations per day did not differ between genotypes.

	WTSED	WTRUN	TGSED	TGRUN
Swim speed (m/s)	0.15 ± 0.02	0.16 ± 0.03	0.11 ± 0.03	0.18 ± 0.03
Wheel rotations/day	n/a	4408 ± 783	n/a	3954 ± 877

**Figure 1:**
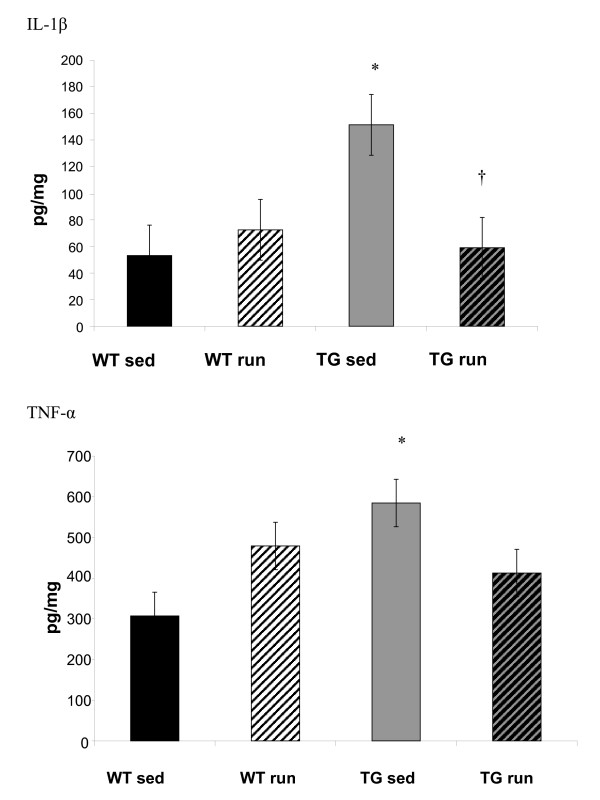
IL-1β is significantly greater in sedentary Tg2576 mice than in WT sedentary mice (p = 0.006). Exercise results in a significantly lower level of IL-1β in the Tg2576 (p = 0.01). The level of IL-1β in exercised Tg2576 mice (TG_RUN_) is no longer distingushable from the WT mouse (WT_SED_). TNF-α is significantly greater in sedentary Tg2576 mice (TG sed) than in WT sedentary mice (p = 0.04). Exercise reduces TNF-α in TG mice (TG run) to a level indistinguishable from the WT (WT_SED_). *Significantly different from sedentary WT † significantly different from sedentary Tg2576.

**Figure 2:**
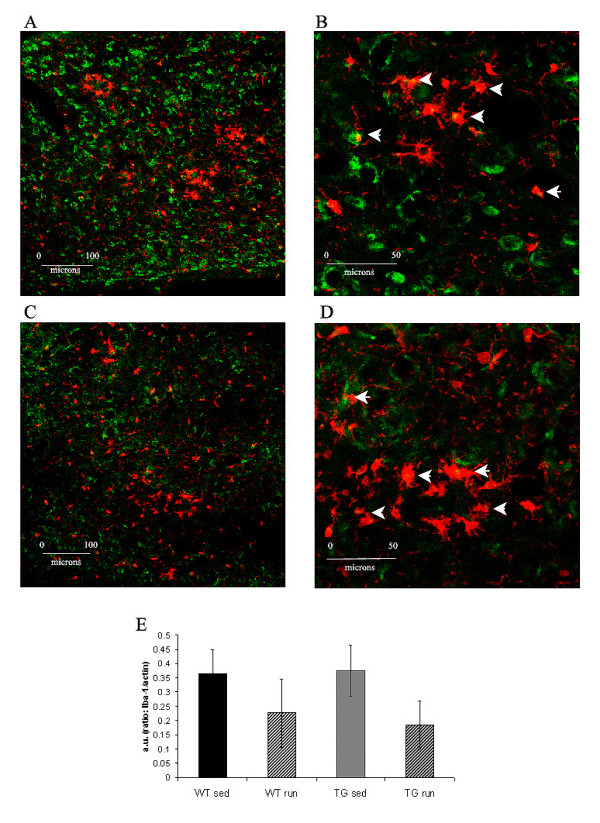
CD11b positive microglia (green immunofluorescence) in TG_SED_(A). Higher magnification reveals some co-labeling with microglial marker Iba-1 (red) (B, arrowheads). CD11b positive glia are present in TG_RUN _(C) and co-labeled with Iba-1 (red) in some cases (D, arrowheads). Overall levels of Iba-1 (normalized to actin) are not significantly different based on condition or genotype (E). High immunoreactivity for Iba-1 in WT is likely due to the advanced age of the animals used.

IL-1β and TNF-α protein were significantly greater in aged sedentary Tg2576 mice (TG_SED_) than in sedentary wildtype (WT_SED_) (IL-1β: p = 0.006; TNF-α: p = 0.04) (Figure 1). Three weeks of exercise decreased the levels of both IL-1β and TNF-α to a point where they no longer significantly differed from the WT_SED_. The IL-1β in exercised Tg2576 mice (TG_RUN_) was significantly lower than in the TG_SED _(p = 0.01)(Figure 1). When we compare current cytokine markers to our previously published behavioral data, the decreased levels of the pro-inflammatory cytokine IL-1β observed in transgenic animals correlate with decreased mean latency to find the escape platform on day 2 of the radial arm version of the Morris water maze (correlation = 0.31, p = 0.001)[49]. We observed CD11b+ microglia in TG_SED _and TG_RUN _animals (Figure 2). Levels of microglial activation, as measured by Iba-1 western blot, did not change with exercise (Figure 2).

**Figure 3:**
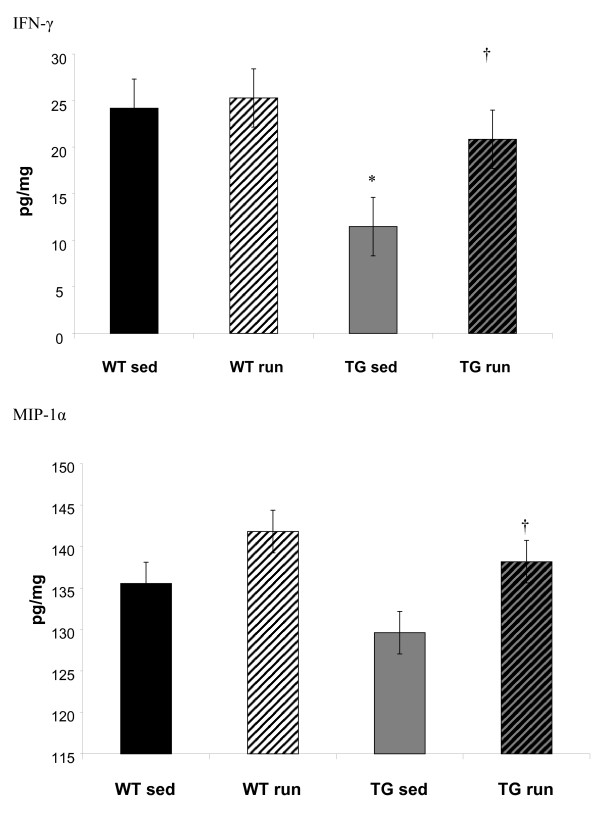
IFN-γ is significantly lower in the Tg2576 sedentary mice that in the WT sedentary mice (p = 0.03). Exercise resulted in increased levels of IFN-γ in the Tg2576 mouse (TG_RUN_) to a level indistinguishable form the WT (WT). MIP-1α demonstrated a trend of being lower in TG_SED _compared to the WT (p = 0.07), but was significantly increased by exercise (TG_RUN_) (p = 0.05). *Significantly different from sedentary WT; † significantly different from sedentary Tg2576.

**Figure 4:**
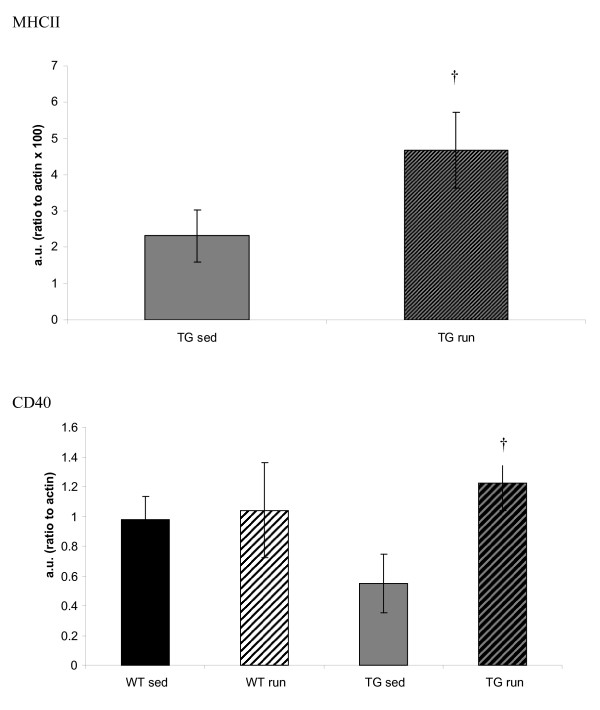
MHC II levels were significantly greater in TG_RUN _than TG_SED _(p = 0.04). CD40 is significantly greater in TG_RUN _compared to TG_SED _(p = 0.008). WT_SED _tended to have greater levels of CD40 than TG_SED_, but this difference failed to achieve significance (p = 0.10). † Significantly different from sedentary Tg2576.

**Figure 5:**
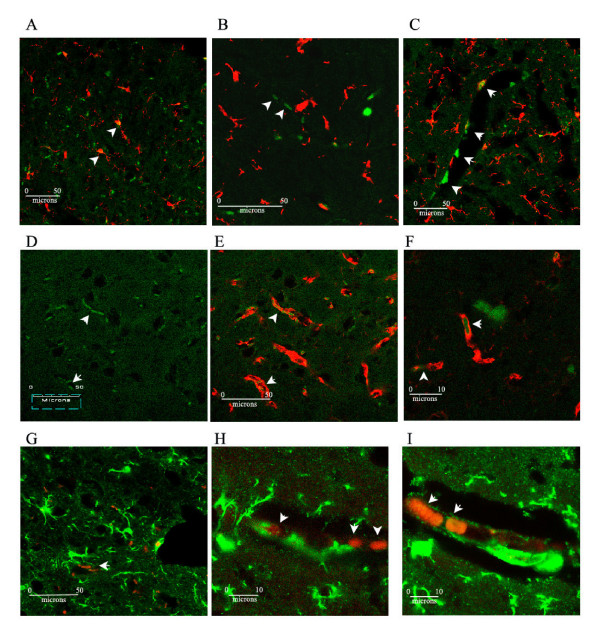
CD11c positive microglia (green immunofluorescence) are present in TG_SED _and colocalize with Iba-1 (arrowheads) but do not appear vascular (A). CD11c labeling in TG_RUN _appeared in cells not labeled by Iba-1 (red) that were linearly arranged, perhaps within or around microvessels. (B, D). Larger vessels had CD11c labeling along the vessel wall, perhaps in the perivascular space (C). Using macrophage markers CD68, we observed microvascular labeling again only in TG_RUN _(D). Double labeling for CD11c (green) and CD68 (red) revealed that CD11c+ cells were adjacent to CD68+ cells in and around vasculature (arrows) (E, F). Using mannose receptor antibody (red), specific for perivascular macrophages, we again observed vascular labeling only in TG_RUN _(G-I). High magnification shows the mannose receptor labeled cells are within vessels (H, I)(arrowheads). Green indicates Iba-1 labeling for microglia in and around vessels (G-I).

In contrast, IFN-γ was significantly lower in aged TG_SED _than in WT_SED _(p = 0.02) and showed a trend to increase in TG_RUN _(p = 0.06) such that it returned to levels similar to the WT (Figure 3). Similarly, protein levels of chemokine MIP1α that tended to be lower in the TG_SED _than the WT_SED _(p = 0.07), were significantly increased in TG_RUN_animals (p = 0.05) to levels similar to those observed in the WT (Figure 3). The MHC II blots confirm that the IFN-γ increase observed is associated with increased antigen presentation in the TG_RUN _animals, who expressed significantly more MHC II than TG_SED _(p = 0.04) (Figure 4). In the TG_RUN _animals, we observed abundant antigen presenting cells, indicated by CD11c (Figure 5B). In TG_RUN_, we observed CD11c+ blood vessels and in individual cells that appeared linearly arranged, as if inside of blood vessels (Figure 5B,C). Vessels labeled strikingly with CD68 (red) in proximity to, but not within the same cells as CD11c (green), suggesting alternatively activated macrophages may be present (Figure 5D–F). Vessels were double labeled for Iba-1 (green) and mannose receptor (red), a marker for perivascular macrophages (Figure 5G–I). No such cells or vessels were observed using the same markers in TG_SED _animals.

The presence of MHCII and CD11c labeled cells indicate antigen presentation. Increased MIP-1α levels suggest T cells and monocytes are being recruited to the brain (Man, 2007). CD40, expressed by the antigen presenting cell communicates with its ligand on T cells. In sedentary TG animals, a trend of reduced expression of CD40 is present compared to WT_SED _(p = 0.10), but there is a significant elevation in CD40 from TG_SED _to TG_RUN _(p = 0.008) (Figure 4). Additionally, alternative activation of macrophages could explain the shift in immune markers observed [29]. Indeed, if we consider all of the cytokine and chemokine changes collectively, along with the conflicting reports of T cell presence and function in the brain [27], alternative activation of macrophages may be a more likely candidate for repair in an AD state [52].

**Figure 6:**
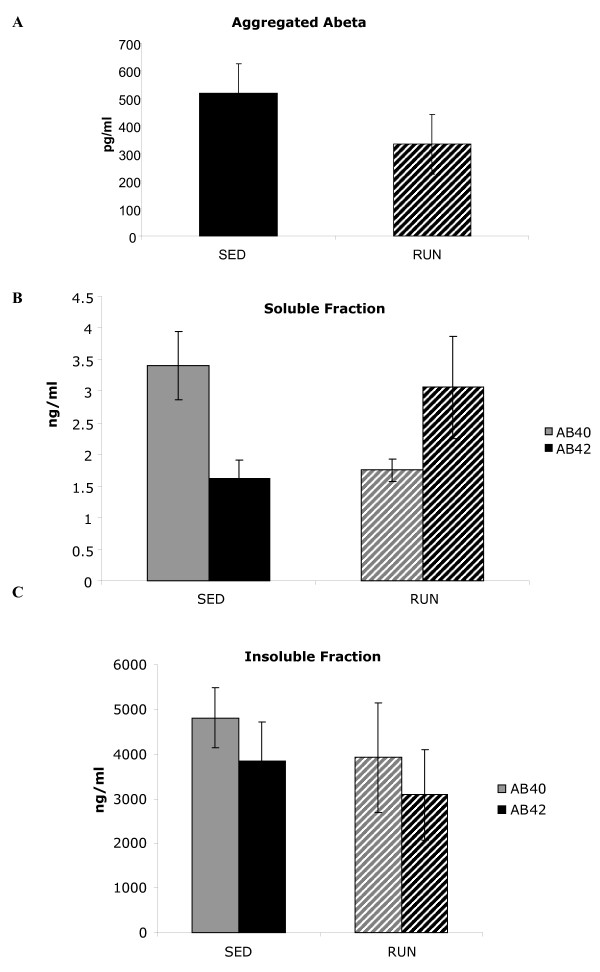
Aβ analysis by multiplex and ELISA. Aggregated Aβ levels are not significantly lower in hippocampus of TG_RUN _compared to TG_SED_, though a 35% decrease is observed in means. (A) Aβ_40 _but not Aβ_42 _is significantly lower in soluble fractions from cortex of TG_RUN _and TG_SED _(p = 0.01)(B). There are no significant differences in insoluble fractions (C).

Finally, we examined whether exercise leads to reduced Aβ in aged Tg2576 mice. We quantified the levels of various Aβ species in cortex by ELISA, and in hippocampus and serum using multiplex technology as well as dot blot. Multiplex revealed no statistically significant differences in Aβ aggregates in hippocampus, though direction of change was to decrease in run (Figure 6A). There was no detectable aggregated Aβ in the serum samples and immunohistochemical staining for plaques appeared similar between TG_SED _and TG_RUN _(data not shown). ELISA of Aβ_42 _in soluble and insoluble fractions from cortex did not statistically differ between TG_SED _and TG_RUN _though we should note that there was a large degree of variation between the RUN animals (Figure 6B–C) Total soluble levels of Aβ_40 _did show a significant decrease (p = 0.01) in theTG_RUN _animals compared to the TG_SED _(Figure 6B). Soluble Aβ is composed of both fibrillar and pre-fibrillar oligomeric species[51]. We used a conformation specific fibrillar antibody to explore changes with exercise in the Tg2576 [39].

**Figure 7:**
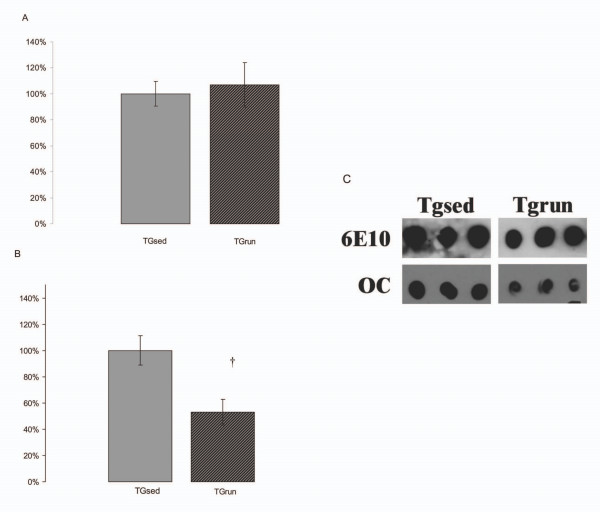
Aβ analysis by dot blot. No differences existed between TG_SED _and TG_RUN _for total Aβ in the soluble fraction of hippocampal samples, evaluated by 6E10 antibody (A). Aβ fibrils, detected by OC antibody, were significantly decreased in TG_RUN _animals compared to TG_SED _(p = 0.01)(B). A representative dot blot is shown (C).

The dot blots of hippocampal lysates are able to selectively probe different soluble Aβ species in a soluble fraction, spun at low speeds. First, we analyzed total Aβ using 6E10 on dot blots. We did not detect any significant differences between SED and RUN (Figure 7A). The OC antibody detects soluble fibrillar Aβ that is conformationally and immunologically distinct [51]. Levels of soluble Aβ fibrils were 40% lower in TG_RUN _compared to TG_SED _as assessed by OC antibody (Figure 7B). This decrease was significant (p = 0.01).

## Discussion

We have demonstrated that physical exercise can shift the immune response in the brain of an AD mouse model. AD is characterized by an innate immune response characterized by microglial activation. Though likely an intended protective mechanism to phagocytose Aβ, these glia are not capable of combating chronic AD pathology [53]. In addition, the cytokines released by these microglia, IL-1β and TNF-α, become cytotoxic when chronically expressed, contributing to the overall neurodegeneration [20,21,24,35,54-56]. We confirmed this immune state in our sedentary aged TG animals. Our findings confirm previous findings that high IL-1β levels are correlated with poor cognitive performance [57] and indicate that exercise induces reductions in TNF-α and IL-1β in the hippocampus of Tg2576 mouse at advanced age.

Recent investigations into bone marrow derived cells that differentiate into microglia in the brain, in concert with studies where microglial phenotype is experimentally altered to an antigen presenting phenotype collectively suggest that if the adaptive immune response is stimulated, Aβ pathology in AD can be reduced and cognition improved [58,59,34,35,38]. The antigen presenting response is characterized by CD11c positive cells, MHC II, and CD40. IFN-γ has been shown to be a stimulus for this microglial phenotype and has also been found to be capable of inducing Aβ clearance [37]. We demonstrate that the aged TG_SED _mouse express lower levels of IFN-γ than the WT and that exercise results in increased IFN-γ and increased markers of the antigen presenting response. In addition to antigen presenting microglia from the local environment, it is also possible that exercise increases recruitment of peripheral monocytes to the brain, where they too can become antigen presenting cells. MIP-1α recruits central and/or peripheral macrophages to the area of plaque deposition in AD and MIP-1α was found to increase in TG_RUN_[60,61]. However, the possibility of peripheral recruitment is in debate, as recent data indicates that much of the evidence in favor of monocyte recruitment to the brain only occurs in the presence of damage to the blood-brain barrier due to the whole-body irradiation used in the original experiments [38,59,62-64]. The integrity of the blood brain barrier is compromised in AD and in the Tg2576 mouse model, however, so we cannot rule out either a peripheral or central source for these microglia [65-67]. Though the origin or the microglia will be a topic for continued investigation, we do demonstrate that the microglia present in the hippocampus after exercise in the aged TG mice exhibit characteristics of the adaptive immune response and antigen presentation.

Another interesting possibility presented by the vascular localization of macrophagic markers observed in TG_RUN _animals is the alternative activation of perivascular cells as macrophages and antigen presenting cells. Though microglia are the initial immune responders in the CNS, perivascular cells may be more efficient at both phagocytosis of antigen and presentation of that antigen than adult microglia [25,68]. In particular, our data revealing extensive mannose receptor labeling in TG_RUN _animals strongly indicates perivascular macrophages are involved in the immune response, as this receptor is not expressed by microglia [31].

We suggest that the antigen being presented is Aβ or an Aβ fragment. Fibrillar Aβ is a highly cytotoxic species that has been shown to adhere to microglia [69,70]. If microglia are stimulated to take on an antigen presenting phenotype, fibrillar Aβ can be cleared from the brain [53]. In the current study, soluble Aβ_40 _and soluble fibrillar Aβ significantly decreased after three weeks of running coincident with the increase in adaptive immune markers and the improvement in behavior [49]. We also observed CD11c adjacent to mannose receptor and CD68 positive cells within the vasculature of TG_RUN_. This presence of perivascular macrophages in conjunction with markers of antigen presentation might be indicative of clearance of Aβ into the periphery, supporting the "peripheral sink" hypothesis, in which Aβ is proposed to efflux from brain into plasma after immunization therapy [71-73]. Indeed, one proposed function of the mannose receptor which we observed in TG_RUN _is antigen transport [31]. Vasilevko et al. (2007) recently showed that direct immunotherapy with anti-Aβ antibodies resulted in decreased diffuse Aβ deposits in brain, but increased Aβ_40 _in plasma [40].

In this study, we present evidence that exercise decreases the chronic pro-inflammatory response well known to associate with AD. The decrease in at least one of the cytokines, IL-1β, correlates with improved ability to solve a water maze task. Unlike ibuprofen and other anti-inflammatory drugs used in AD treatment, which decrease pro-inflammatory markers, the current study provides evidence that exercise not only decreases pro-inflammatory markers like IL-1β and TNF-α, but also increases adaptive inflammatory markers IFN-γ, CD40, MHC II, CD11c, and MIP-1α. We observed multiple macrophage markers (CD68, mannose receptor) in and around the vasculature of TG_RUN _animals. Further, we have shown that exercise decreases soluble Aβ_40 _and soluble fibrillar Aβ. We support the hypothesis put forward by Butovsky et al (2006) that the innate immune response in AD becomes dysfunctional after chronic activation, but that an initiation of the adaptive or alternative immune response may reduce pathology [35]. The decrease in fibrillar Aβ and improvement in behavior observed in our investigations of the Tg2576 mouse at 17–19 months of age suggests that physical exercise can trigger this immune shift in late stages of AD, leading to cognitive improvement [49].

## Conclusion

Our data suggests that exercise intervention may pave the way for successful Aβ clearance even in late stages of AD. We suggest that exercise not only decreases neurotoxic cytokines, but also increases Aβ clearance through its induction of an adaptive or alternative immune response. In summary, our investigation opens a potential avenue for behavioral interventions that would be complementary to long term NSAIDs or anti-inflammatory drugs.

## List of abbreviations

WT: Wildtype; TG: Transgenic; SED: Sedentary; AD: Alzheimer's disease; IL: Interleukin; TNF: Tumor necrosis factor; MHC: Major histocompatibility complex; IFN: Interferon; MIP: Macrophage inflammatory protein; CNS: Central nervous system; NSAIDS: Non-steroidal anti-inflammatory drugs.

## Competing interests

CGG is a consultant for Kinexis, Inc. The remaining authors declare that they have no competing interests.

## Authors' contributions

KEN designed and implemented the investigation and was responsible for data analysis, interpretation, and preparation of the manuscript. WWP performed the for Aβ dot blots. AIP aided in study design and preparation of tissue for histology. DHC provided Aβ ELISA data and contributed to the direction of the investigation and data interpretation. CGG created the unique antibodies used to assess Aβ in the dot blot analysis. CWC contributed to the writing of the manuscript and the data interpretation.
